# Sodium/*myo*-Inositol Transporters: Substrate Transport Requirements and Regional Brain Expression in the TgCRND8 Mouse Model of Amyloid Pathology

**DOI:** 10.1371/journal.pone.0024032

**Published:** 2011-08-26

**Authors:** Daniela Fenili, Ying-Qi Weng, Isabelle Aubert, Mark Nitz, JoAnne McLaurin

**Affiliations:** 1 Department of Laboratory Medicine and Pathobiology, University of Toronto, Toronto, Ontario, Canada; 2 Brain Sciences, Sunnybrook Research Institute, University of Toronto, Toronto, Ontario, Canada; 3 Department of Chemistry, University of Toronto, Toronto, Ontario, Canada; University of South Florida, United States of America

## Abstract

Inositol stereoisomers, *myo*- and *scyllo*-inositol, are known to enter the brain and are significantly elevated following oral administration. Elevations in brain inositol levels occur across a concentration gradient as a result of active transport from the periphery. There are two sodium/*myo*-inositol transporters (SMIT1, SMIT2) that may be responsible for regulating brain inositol levels. The goals of this study were to determine the effects of aging and Alzheimer's disease (AD)-like amyloid pathology on transporter expression, to compare regional expression and to analyze substrate requirements of the inositol transporters. QPCR was used to examine expression of the two transporters in the cortex, hippocampus and cerebellum of TgCRND8 mice, a mouse model of amyloid pathology, in comparison to non-transgenic littermates. In addition, we examined the structural features of inositol required for active transport, utilizing a cell-based competitive uptake assay. Disease pathology did not alter transporter expression in the cortex or hippocampus (p>0.005), with only minimal effects of aging observed in the cerebellum (SMIT1: F_2,26_ = 12.62; p = 0.0002; SMIT2: F_2,26_ = 8.71; p = 0.0015). Overall, brain SMIT1 levels were higher than SMIT2, however, regional differences were observed. For SMIT1, at 4 and 6 months cerebellar SMIT1 levels were significantly higher than cortical and hippocampal levels (p<0.05). For SMIT2, at all three ages both cortical and cerebellar SMIT2 levels were significantly higher than hippocampal levels (p<0.05) and at 4 and 6 months of age, cerebellar SMIT2 levels were also significantly higher than cortical levels (p<0.05). Inositol transporter levels are stably expressed as a function of age, and expression is unaltered with disease pathology in the TgCRND8 mouse. Given the fact that *scyllo*-inositol is currently in clinical trials for the treatment of AD, the stable expression of inositol transporters regardless of disease pathology is an important finding.

## Introduction

*scyllo*-Inositol is currently in human clinical trials for the treatment of patients with mild to moderate Alzheimer's disease (AD; www.clinicaltrials.gov). This stemmed from preclinical studies, in which *scyllo*-inositol was shown to be an effective treatment for AD-like amyloid pathology and cognitive deficits in TgCRND8 mice [1], [2]. TgCRND8 mice show many of the hallmark features of AD, including an increase in cerebral Aβ levels, Aβ aggregation and plaque deposition, along with cognitive deficits as the disease advances [3]. *scyllo*-Inositol treatment significantly improved spatial memory, synaptic function and survival rates in TgCRND8 mice [1]. These positive effects occurred both in animals given *scyllo*–inositol prophylactically, before the visible onset of symptoms, and therapeutically, once symptoms had fully developed [1], [2]. Gas chromatography/mass spectrometry results found *ad libitum scyllo*-inositol treatment increased *scyllo*-inositol levels within the brain 7-fold [2] and magnetic resonance spectroscopy also demonstrated a 2 to 3-fold increase in brain *scyllo*-inositol levels [4] suggesting that the beneficial effects are centrally induced.

*scyllo*-Inositol is found endogenously in the body and is the second most abundant inositol stereoisomer [5], [6]. The brain levels of *myo*- and *scyllo*-inositols are 100-fold greater than those found in the periphery [5], [7]. This indicates that active transport, in addition to simple diffusion, is required for the regulation of brain inositol levels. Active transport of inositol stereoisomers across cellular membranes including cells at the blood-brain and blood-CSF barriers, occurs via inositol transporters. Two transporters, SMIT1 and SMIT2, are important for regulating brain and peripheral inositol levels, by co-transporting two sodium ions along the concentration gradient, to generate enough energy to actively transport *myo*-inositol [8]–[12]. While both transporters are expressed in the brain [11], [13], [14], there is limited information on the regional expression of these transporters and no data on expression changes with various disease pathologies, including AD. In addition, there is little information on age-induced changes to transporter expression or comparative data on expression of SMIT1 and SMIT2. To answer these questions, transporter mRNA levels were examined in three brain regions: the cortex, hippocampus and cerebellum, as a function of age, genotype and Aβ/amyloid related pathology. These brain regions were chosen as the cortex and hippocampus are most affected in AD while the cerebellum is only affected very late in the disease. Expression was examined in both TgCRND8 mice and non-transgenic (Tg) littermates at 2, 4 and 6 months of age. These ages were selected because they correspond to pre-plaque deposition, mid-stage and advanced AD-like amyloid pathology in TgCRND8 mice [3]. Further, transport assays were conducted to determine the inositol structural features required to decrease *myo* or *scyllo*-inositol transport through SMIT1/2. The aims of this study were to extend our knowledge of SMIT1 and SMIT2 expression as a function of age and disease pathology and to better understand substrate transport through these transporters. Both of these experimental aims were achieved.

## Results

### Inositol transporter expression as a function of age and progressive amyloid deposition

**Figure 1:**
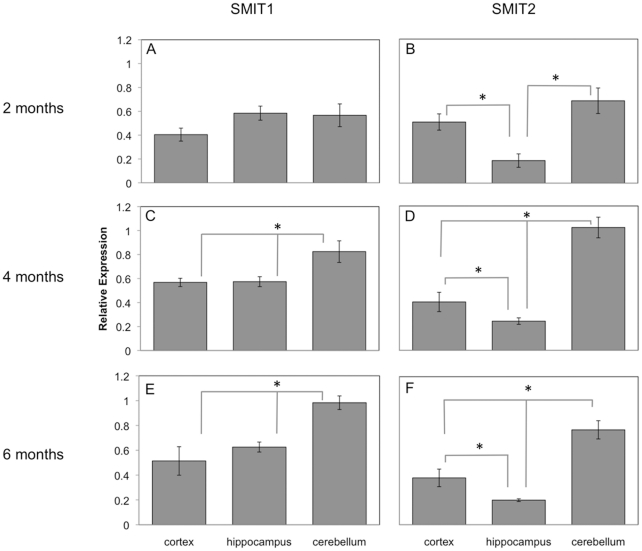
A comparison of regional expression for each of the inositol transporters. A comparison of the regional expression patterns of SMIT1 (**A, C, E**) and SMIT2 (**B, D, F**) in the brain at 2 months (**A, B**), 4 months (**C, D**) and 6 months of age (**E, F**) showed a distinct pattern of expression. SMIT1 levels were significantly higher in the cerebellum than in the cortex or hippocampus at 4 and 6 months of age. SMIT2 levels were significantly higher in the cortex and cerebellum, compared to the hippocampus at all three time points and significantly higher in the cerebellum, compared to the cortex, at 4 and 6 months of age. (n = 10 animals/group; p<0.05).

*scyllo*- and *myo*-Inositol accumulate in the brain, at levels 100-fold higher than those in the periphery [5], [7], through active transport. There are two sodium *myo*-inositol transporters that have been reported in the literature, SMIT1 and SMIT2, both of which are expressed in the brain [11], [13], [14]. Normal brain aging has been linked with neuronal cell loss, an increase in gliosis and ultimately an increase in both *myo*- and *scyllo*-inositol as shown by magnetic resonance spectroscopy [15], [16]. This increased inositol signal may be due to increased transporter activity or expression. Therefore, one goal of these experiments was to examine whether the levels of these transporters change as a function of age. The mRNA expression of the two inositol transporters was examined at 2, 4 and 6 months of age in both TgCRND8 mice and non-Tg littermates in the cortex, hippocampus and cerebellum. Significant age effects on transporter expression were observed in the cerebellum of both groups (SMIT1: F_2,26_ = 12.62; p = 0.0002; SMIT2: F_2,26_ = 8.71; p = 0.0015), but not in the cortex or hippocampus (p>0.05). In both TgCRND8 mice (Figure 1) and non-Tg littermates (data not shown), 2 month SMIT1 and SMIT2 levels were significantly lower than 4 and 6 month levels (p<0.05). More specifically, in TgCRND8 mice, 2 month, cerebellar SMIT1 levels were significantly lower than 4 and 6 month levels (Figure 1A, C, E; p<0.05) and 2 month, cerebellar SMIT2 levels were significantly lower than 4 month levels (Figure 1B, D; p<0.05). In non-Tg littermates, 2 month, cerebellar SMIT1 and SMIT2 levels were significantly lower than 6 month levels (data not shown; p<0.05). These regional differences, for the effects of age on expression, may reflect the continual postnatal maturation of the cerebellum [17], which does not occur in the cortex or hippocampus.

A comparison of SMIT1 and SMIT2 expression between TgCRND8 mice and non-Tg littermate mice, at 2, 4 and 6 months of age, was used to determine the effects of AD-like amyloid pathology on transporter expression. No significant differences in SMIT1 or SMIT2 expression were observed between TgCRND8 mice and non-Tg littermates at all ages and in all regions, indicating that increasing amyloid pathology does not alter the expression of the transporters (p>0.05; data not shown).

### Regional brain inositol transporter expression levels

The expression of SMIT1 and SMIT2 were quantified in the cortex, hippocampus and cerebellum (Figure 1). These three brain regions are affected to varying degrees by Aβ plaque deposition in AD [18]. The earliest plaque deposition is observed in the cortex and hippocampus, while deposition in the cerebellum occurs very late in the disease [18]. Both transporters were expressed in all three brain regions examined, however regional differences in expression for each transporter were observed. For both transporters, a significant correlation between brain region and transporter expression levels was found (Figure 1; SMIT1: F_2,86_ = 14.86, p<0.0001; SMIT2: F_2,86_ = 55.04, p<0.0001). For SMIT1, no significant differences in regional expression were observed at 2 months (Figure 1A), but at 4 and 6 months, cerebellar SMIT1 levels were significantly higher than cortical and hippocampal levels (Figure 1C, E; p<0.05). For SMIT2, at all three ages both cortical and cerebellar SMIT2 levels were significantly higher than hippocampal levels (Figure 1B, D, F; p<0.05) and at 4 and 6 months of age, cerebellar SMIT2 levels were also significantly higher than cortical levels (Figure 1D, F; p<0.05).

### Structural requirements for inositol transport

**Figure 2:**
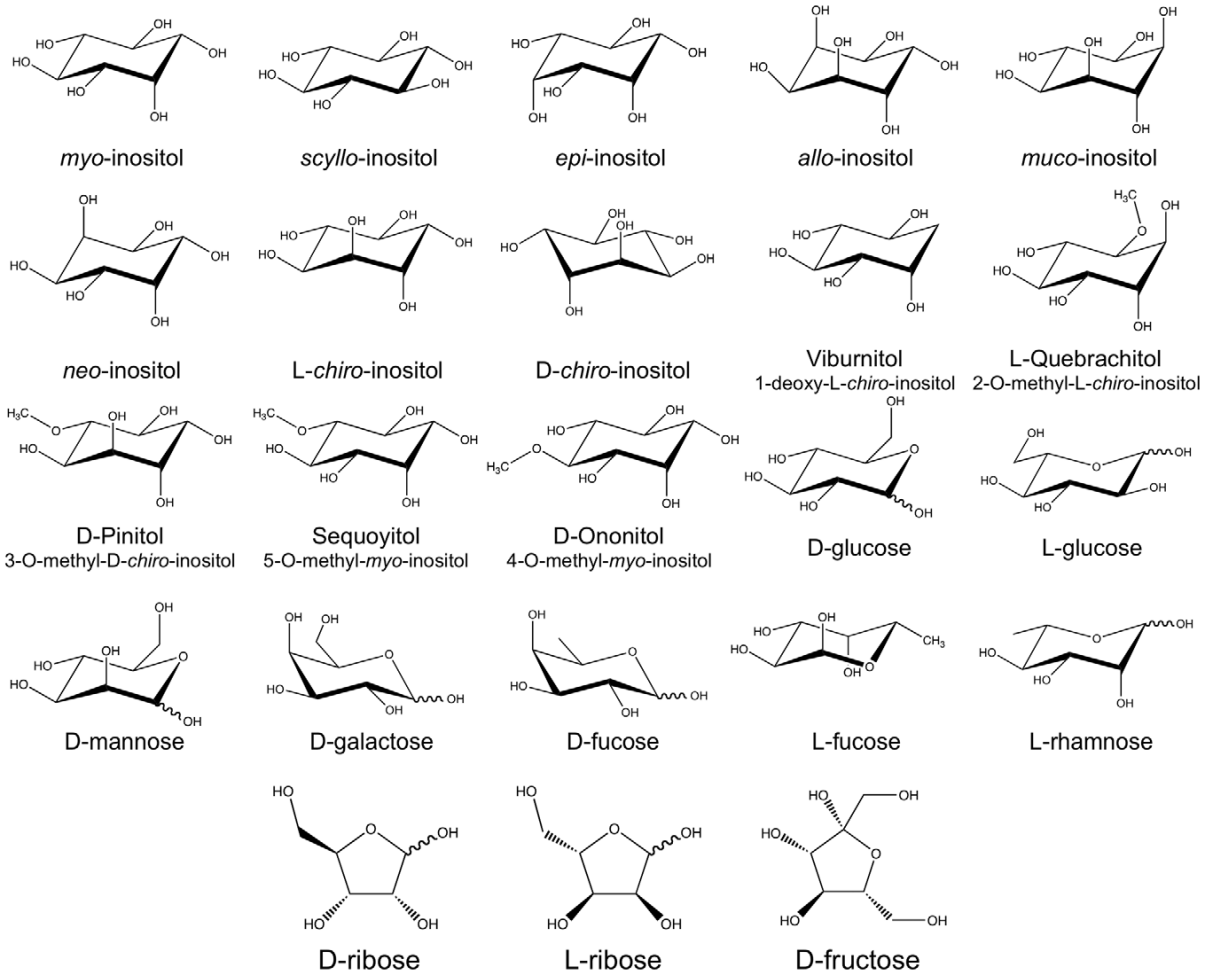
Structures of the inositol stereoisomers, derivatives and related compounds. The structures of the inositol stereoisomers, derivatives and related compounds used for the initial competitive transport assays.

**Figure 3:**
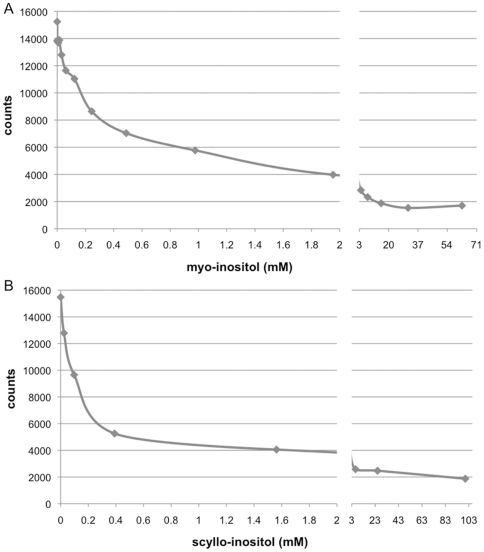
*myo*-Inositol and *scyllo*-inositol transport in HEK293 cells. *myo*-Inositol testing was conducted in HEK293 cells, by incubating cells for 15 minutes in medium containing 100 µM of *myo*-inositol-(2-^3^H) in the presence of increasing concentrations of cold *myo*-inositol (**A**). Addition of 10 mM of cold *myo*-inositol, the concentration used in the competitive transport assays, resulted in a near maximal inhibition of *myo*-inositol-(2-^3^H) transport in these cells. *scyllo*-Inositol testing was conducted over a period of 3 hours in these cells, due to slower uptake rates (**B**). Once again, 10 mM of cold *scyllo*-inositol produced near maximal inhibition of *scyllo*-inositol-(2-^3^H). (n = 3 wells per concentration).

Although SMIT1/2 transport of various inositol stereoisomers has been reported, the structural requirements for transport have not been examined. To define these parameters, transport of *myo*- or *scyllo*-inositol-(2-^3^H) was examined in the presence of 15 inositol isomers, 6 hexoses and 2 pentose sugars which may serve as competive substrates, inhibitors or allosteric effectors on inositol transport (Figure 2). We first examined the *myo*- and *scyllo*-inositol transport kinetics of our cell system (Figure 3). The transport of *myo*- or *scyllo*-inositol-(2-^3^H) (100 µM) was assayed in the presence of increasing concentrations of cold *myo*- or *scyllo*-inositol. The concentration dependence of *myo*-inositol saturated near 10 mM of cold *myo*-inositol producing a near maximal inhibition of *myo*-inositol-(2-^3^H) transport, while half-maximal inhibition was observed at 200 µM of cold *myo*-inositol (Figure 3A). As was seen for *myo*-inositol, 10 mM of cold *scyllo*-inositol produced near maximal inhibition of *scyllo*-inositol-(2-^3^H) transport and half maximal inhibition was observed at 200 µM cold *scyllo*-inositol (Figure 3B). However, while the *myo*-inositol transport experiment was conducted using a 15 minute incubation window, the *scyllo*-inositol experiment required the incubation to be extended to 3 hours in order to produce similar uptake of *scyllo*-inositol. These results highlight an apparent slower uptake rate for *scyllo*-inositol by the transporters. Our half maximal inhibition values for *myo*-inositol agree well with the range previously reported in the literature; *myo*-inositol K_m_ = 55–117 µM for SMIT1 and K_m_ = 120–348 µM for SMIT2 [9], [10], [19]–[24].

**Figure 4:**
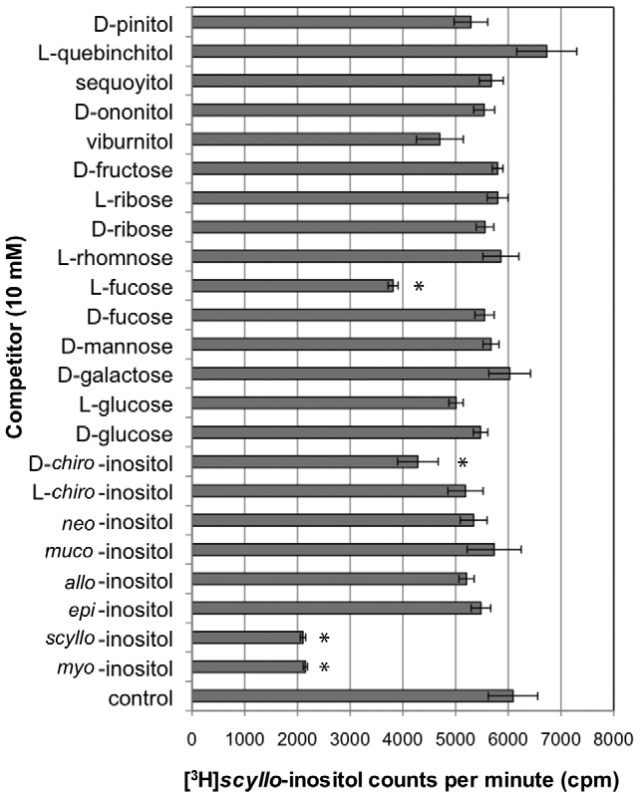
*scyllo*-Inositol-(2-^3^H) transport in HEK 293 cells. *scyllo*-Inositol transport in the presence of inositol and sugar isomers was examined by measuring *scyllo*-inositol-(2-^3^H) transport in the presence of each of those potential substrates. For the control experiment, *scyllo*-inositol-(2-^3^H) transport was quantified in the absence of any added compounds. Cells were incubated for 3 hours in medium containing 100 µM of *scyllo*-inositol-(2-^3^H) in the presence or absence of 10 mM of potential inositol or sugar isomer transport competitors and the resulting radioactivity in the cells was measured (n = 3 wells per variable; * = p<0.05).

**Figure 5:**
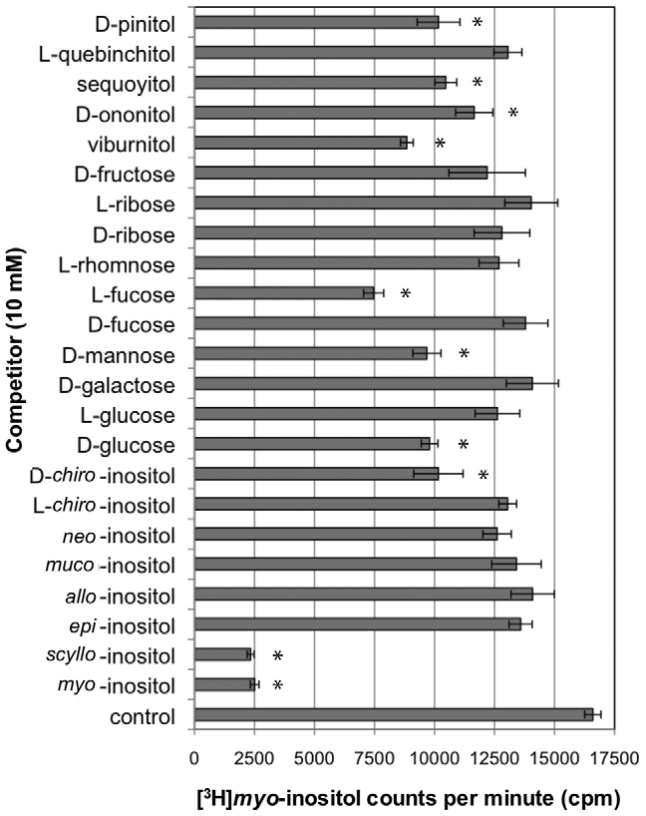
*myo*-Inositol-(2-^3^H) transport in HEK 293 cells. *myo*-**inositol** transport was examined by measuring uptake of *myo*-inositol-(2-^3^H) (100 µM) in the presence or absence of 23 different inositols or sugars (10 mM) over a 3 hour incubation period, after which radioactivity was measured. For the control experiment, *myo*-inositol-(2-^3^H) transport was quantified in the absence of any competitive substrates. (n = 3 wells per variable; * = p<0.05).

An examination of *scyllo*-inositol-(2-^3^H) transport, in the presence or absence of the 23 polyols, showed *myo*-, *scyllo*-, D-*chiro*-inositol and L-fucose to significantly depress *scyllo*-inositol-(2-^3^H) transport (Figure 4; F_23,48_ = 14.47, p<0.05). In contrast, when *myo*-inositol-(2-^3^H) transport was tested in the presence or absence of the 23 polyols, in addition to those identified for *scyllo*-inositol, D-glucose, D-mannose, viburnitol, sequoyitol, D-ononitol and D-pinitol also significantly depressed *myo*-inositol-(2-^3^H) transport (Figure 5; F_23,48_ = 18.46, p<0.05). This difference in experimental findings likely stems from differences in the relative transport rates of *myo*- and *scyllo*-inositol by SMIT1/2 and differences in specificity of the transporters, which would result in different observed inhibition profiles. Overall, our results are in agreement with previous studies that identified *myo*-, *scyllo*-, D-*chiro*-inositol, D-glucose and D-pinitol as substrate competitors [9], [10], [19], [22], [24].

**Figure 6:**
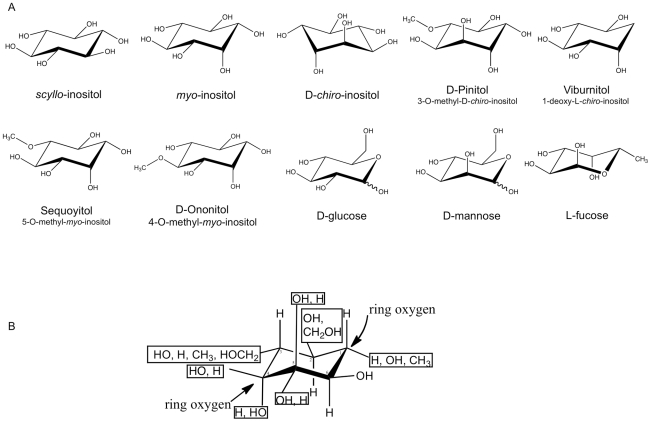
Basic structural model for SMIT1/2 substrate transport competitors. The structures of the substrates which competed for SMIT1/2 *myo*-inositol-(2-^3^H) transport (**A**), along with the structures of substrates that did not compete, were used to design a basic structural model for SMIT1/2 substrate transport competitors (**B**).

A comparison of the structural features of the compounds, which depressed *myo*-inositol, allowed the design of a model of the structural requirements for inhibition of transport through SMIT1/2 (Figure 6). With the panel of potential substrates tested, this model is best used to identify compounds that will not be recognized by the transporters, as additive effects between the substituents cannot be determined. All of the active compounds identified have equatorially positioned side chains at carbons 1, 2, 3 and 6, while the substituents at carbons 4 and 5 can be in either orientation. In addition, a comparison of the substrate competitors shows that the side group at position 1 can be either a hydroxyl, hydrogen, or methyl group and the carbon can be exchanged for a ring oxygen atom and maintain activity in the transport assay. At position 2 either a hydroxyl or a methoxy side chain are tolerated. At position 3, the hydroxyl group can be exchanged for a hydrogen, methyl or methoxyl group, while at position 6, the side chain must remain a hydroxyl group. At positions 4 and 5, either a hydroxyl or a hydrogen group can be placed in either orientation without disrupting the substrates competitive nature and the carbon at position 4 can be exchanged for a ring oxygen atom.

### Substrate recognition

We examined the recognition of the enantiomers D-*chiro*-inositol, which depressed *scyllo*/*myo*-inositol transport through SMIT1/2, and L-*chiro*-inositol, which had no effect, in greater detail. To determine whether this enantiomeric change in structure results in a complete lack of recognition by the transporter or decreased transporter affinity, the effect of increasing concentrations of each enanitomer on transport was examined. L-*chiro*-inositol competed for *myo*-inositol-(2-^3^H) transport only at the highest concentrations assayed (160 mM), consistent with our model and the previous assay carried out using 10 mM L-*chiro*-inositol. In contrast, D-*chiro*-inositol demonstrated a dose-dependent inhibition of *myo*-inositol transport (Figure S1). These results confirm the transporters' selectivity in the enantiomer of *chiro*-inositol recognized.

Based on our initial substrate transport studies L-fucose decreases the rate of *myo*-inositol transport. Further analysis confirmed this result showing that L-fucose reduced *myo*-inositol-(2-^3^H) transport in a concentration-dependent manner (Figure S2A). To determine if L-Fucose was a competitive substrate for SMIT or simply an inhibitor of the transporters the uptake of L-fucose-(5,6-^3^H) was measured. In these experiments no reduction in cell associated L-fucose-(5,6-^3^H) uptake was observed with any of the inositol substrates tested (Figure S2B) and no changes in radioactivity were observed in the presence of increasing concentrations of cold L-fucose (Figure S2C). Therefore, we propose that L-fucose is a SMIT1/2 transport inhibitor as has been previously proposed [9], [25].

**Figure 7:**
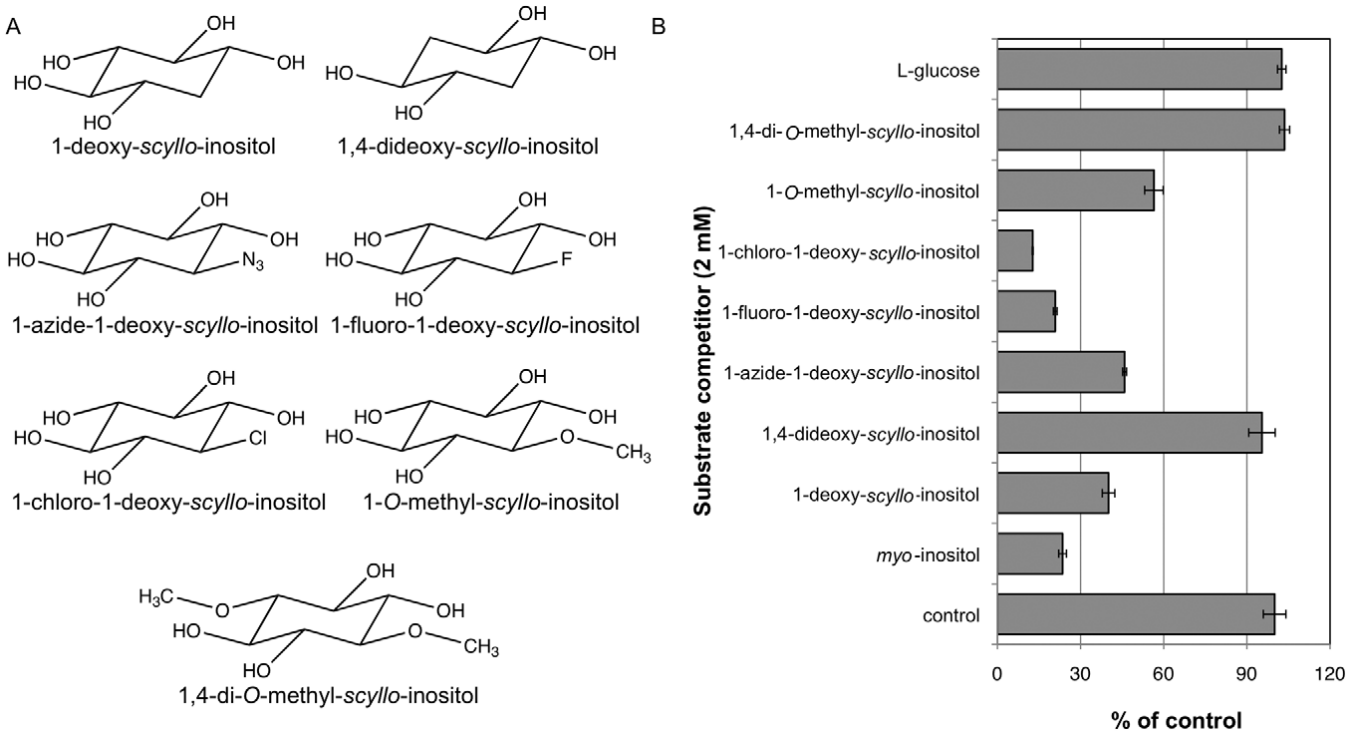
Transport of *scyllo*-inositol derivatives by SMIT1/2. Further testing of SMIT1/2 myo-inositol transport inhibition was conducted using derivatives of *scyllo*-inositol, in which one or two hydroxyl groups were removed or substituted (**A**). For the control experiment, *myo*-inositol-(2-^3^H) transport was quantified in the absence of any competitors. An analysis of *myo*-inositol-(2-^3^H) transport in the presence of each of the compounds, found monodeoxy- but not dideoxy-*scyllo*-inositol competed for *myo*-inositol-(2-^3^H) transport (**B**). Single hydroxyl group substitutions were tolerated in the order chlorine>fluorine>nitrate>hydroxy methyl. (n = 3 wells per variable).

The SMIT1/2 basic recognition model was further tested and refined using a set of *scyllo*-inositol derivatives initially designed for examination of the effects of *scyllo*-inositol derivatives on Aβ fibrillogenesis [26], [27]. The use of these compounds allowed us to address further the hydrogen-bonding requirements and steric interactions between a given compound and the transporters. Seven compounds were tested, in which either: one or two hydroxyl groups were substituted with hydrogen or methoxy, or one hydroxyl group was replaced with a fluorine, azide, or chlorine (Figure 7A). Transporter recognition was observed following the substitution of one hydroxyl group on *scyllo*-inositol with a hydrogen atom or a hydroxy methyl group, however, the substitution of two hydroxyl groups removed any recognition of the compound by the transporters (Figure 7B). The finding that 1,4-di-*O*-methyl-*scyllo*-inositol was not transported is in agreement with the SMIT1/2 model and suggests steric interference of the bulky hydroxy methyl groups. Surprisingly, 1,4-dideoxy-*scyllo*-inositol was also not recognized by the transporters, suggesting that five hydroxyl groups or hydrogen bonding pairs are necessary for transporter interaction. When the effects of substituting one hydroxyl group were further examined, both 1-chloro- and 1-fluoro-1-deoxy-*scyllo*-inositol competed as readily as *myo*-inositol for active transport, while the hydrogen, azide and hydroxy methyl substitutions were less effective (Figure 7B). These results suggest that single small polar or non-polar substitutions on the *scyllo*-inositol scaffold are tolerated but larger substitutions such as methoxy or azide compromise transporter recognition.

## Discussion

*scyllo*-Inositol is present endogenously in both the body and the brain [5], [6], with brain levels 100-fold higher than those in the periphery [5], [7]. This points to the importance of active transport for *scyllo*-inositol accumulation within the brain. Oral administration of *ad libitum myo*- or *scyllo*-inositol to mice increased the corresponding levels in the brain [2]. Both TgCRND8 mice and their non-Tg littermates showed a significant increase in *scyllo*-inositol levels in the brain following *ad libitum* administration, with no significant differences observed in baseline or treatment-induced brain inositol levels between the two groups [2]. In the present study, no significant differences in SMIT1 or SMIT2 expression profiles were found between TgCRND8 mice and their non-Tg littermates at any of the ages examined. Since the three time points chosen correspond to pre-plaque deposition, early plaque deposition and an advanced stage AD-like amyloid phenotype [3], we can conclude that neither age nor amyloid pathology alters SMIT1 or SMIT2 expression. A stable expression pattern, irrespective of pathology, is an important factor for successful *scyllo*-inositol treatment.

A comparison of SMIT1 versus SMIT2 expression in subregions of the brain was also conducted. Although it is known that both inositol transporters are expressed in the brain [11], [13], [14], our study found differences in the relative expression levels of the two transporters across brain regions that are affected in AD. Overall, expression in the brain of SMIT1 was higher than SMIT2. SMIT1 expression was observed in the order cerebellum>cortex = hippocampus while SMIT2 expression was also highest in the cerebellum>cortex>hippocampus. Both transporters were expressed in all the brain regions examined.

The specificity and structural requirements for transport by SMIT1/2 has not been previously investigated. To investigate the recognition of inositol and sugars by the transporters a substrate transport study was conducted. Substrate transport of labeled *myo*- and *scyllo*-inositol showed transport of *scyllo*-inositol to be substantially slower despite the recognition of both substrates with similar affinity (IC_50_ ∼200 µM). An analysis of 23 different inositols and sugars was conducted. Our findings indicate that a number of substrates inhibit *scyllo*- and *myo*-inositol-(2-^3^H) transport. This inhibition could be due to direct competition for transport, active inhibition of the transporters or through allosteric inhibition. Specifically the SMIT1/2 transporters were found to recognize D-*chiro*-inositol, viburnitol, D-glucose, D-mannose, sequoyitol, D-ononitol and D-pinitol all to lesser extents than *myo*- or *scyllo*-inositol. Previously an equal preference for *myo*- and *scyllo*-inositol was shown in *Xenopus* oocytes transfected with canine SMIT1 [9]. SMIT1/2 transport of D-*chiro*-inositol [10], [19], [22], [24] and SMIT2 transport of D-glucose and D-pinitol have been previously reported [24]. We confirm previous observations of SMIT2 preference of these stereochemistries by the transporters [19]. The HepG2 human liver cell-line was shown to preferentially transport D-*chiro*-inositol and D-glucose over L-stereoisomers [19]. An additional substrate, L-fucose, has been previously identified as a competitor for *myo*-inositol transport [9], [25]. The present observations that *myo*-inositol and increasing concentrations of cold L-fucose did not reduce cell associated L-fucose-(5,6-^3^H) levels, allows us to conclude that L-fucose is a SMIT1/2 transport inhibitor.

To further define transporter selectivity, novel derivatives of *scyllo*-inositol, containing single or double hydroxyl group substitutions were examined for transport. One or two hydroxyl groups on *scyllo*-inositol were substituted with chlorine, fluorine, hydrogen, azide or hydroxy methyl groups and transport competition in relation to tritiated *myo*-inositol was examined. Single substitutions competed against tritiated *myo*-inositol transport, with chlorine and fluorine substitutions competing against tritiated *myo*-inositol transport as efficiently as *myo*- or *scyllo*-inositol. However, double substitutions were not recognized as efficiently, highlighting the apparent specificity of the transporters.

In conclusion, inositol transporter expression in the brain was unaltered by amyloid disease pathology, as determined by comparing SMIT1 versus SMIT2 expression profiles in TgCRND8 mice. Overall, SMIT1 expression in the brain was higher than SMIT2, with equivalent expression in the cortex and hippocampus. Expression of both transporters was observed in all the brain regions examined and regional expression profiles were unique. When the inhibition of substrate transport was examined, SMIT1/2 transported *scyllo*-inositol as expected, although at a slower uptake rate than *myo*-inositol. The findings of this study suggest that the beneficial effects seen in the preclinical studies on the efficacy of *scyllo*-inositol in a Tg mouse model of AD can be attributed to the expression of the SMIT1/2 transporters in brain regions susceptible to AD pathology and the steady transport of *scyllo*-inositol to those regions to target AD pathology. These results are particularly important as the intent to advance *scyllo*-inositol to phase III trials was announced after the release of the Topline summary results of the phase II study [28], [29].

## Materials and Methods

### Ethics Statement

All experiments were performed according to the Canadian Council on Animal Care guidelines (Protocol #: 20008707).

### Materials

All reagents were purchased from Sigma (St. Louis, MO, USA) unless otherwise noted. *epi*-Inositol, *allo*-inositol and cold *scyllo*–inositol were acquired from Transition Therapeutics Inc. (Toronto, Ontario, Canada). Viburnitol (1-D-3-deoxy-*myo*-inositol; Cat #: FC-041), D-Ononitol (1-D-4-O-methyl-*myo*-inositol; Cat #: FC-040), Sequoyitol (5-O-methyl-*myo*-inositol; Cat #: FC-047) and D-Pinitol (3-O-methyl-D-*chiro*-inositol; Cat #: FC-026) were purchased from Industrial Research Ltd. (Lower Hutt, New Zealand). *myo*-Inositol-(2-^3^H) (3 µCi/mL; Cat #: ART 0116), *scyllo*-inositol-(2-^3^H) (3 µCi/mL; Cat #: ART 0264) and L-fucose-(5,6-^3^H) (3 µCi/mL; Cat #: ART 0106A) were purchased from American Radiolabeled Chemicals Inc. (St. Louis, MO, USA).

### Mice

TgCRND8 mice were maintained on an outbred C3H/C57Bl6 background. These mice over express the human amyloid precursor protein gene containing both the Swedish (KM670/671NL) and Indiana (V717F) mutations under control of the Syrian hamster prion gene promoter [3]. Mice were kept on a 12-hour light/dark cycle and given water and standard rodent chow *ad libitum*. To quantify inositol transporter expression, TgCRND8 mice and non-Tg littermates at 2, 4 and 6 months of age were anesthetized with pentobarbital, transcardially perfused with cold PBS-heparin, after which, the brains were removed and the cortex, hippocampus and cerebellum dissected for RNA isolation.

### Quantification of inositol transporter expression

QPCR was performed using an Applied Biosystems 7500 Real-Time PCR system and a SYBR® GreenER™ qPCR SuperMix Universal kit (Invitrogen, Cat #: 11762). Primers were designed using the Beacon Designer 7.5 software program for Mac OS X and are listed in Table 1. Tissue RNA was isolated using phenol/chloroform extraction and the sample concentrations determined using a NanoDrop™ Spectrometer (Thermo Scientific, Wilmington, USA). RNA samples were treated with DNase I (Fermentas, Cat #: EN0521) to remove any genomic DNA. RNase inhibitor (Fermentas, Cat #: EO0381) was added to the reaction mixture to prevent RNA degradation during DNase I treatment. The RNA concentration was determined and for each sample, 3 cDNA reactions were performed using a SuperScript III First-Strand Synthesis SuperMix for qRT-PCR kit (Invitrogen, Cat #: 11752). Following reverse transcription, residual RNA was removed through *E. coli* RNase H treatment and the cDNA concentrations normalized. For the QPCR reaction, 10 ng of cDNA was used per well of a 96-well plate. A 2 month, non-tg, kidney, with ideal 260/280 and 260/230 ratios was selected for use as a between plate control. The genes TATA-box binding protein (Tbp) and Glyceraldehyde 3-phosphate dehydrogenase (Gapdh) were selected as control genes, following a scan of the National Center for Biotechnology Information (NCBI), Gene Expression Omnibus website to confirm stable expression in all three of the brain regions examined in this study. For analysis, relative quantification values were normalized to the control genes, inter-plate calibrators and variations in primer efficiencies, then adjusted to an average expression level of 1 using the MultiD, GenEx software program (genex.gene-quantification.info/) for analysis.

**Table 1:** QPCR primers.

Protein Name	Gene	Accession #	Primers
TATA-box Binding Protein	Tbp	NM_013684	Forward: GCC TTC CAC CTT ATG CTC AG Reverse: GAG TAA GTC CTG TGC CGT AAG
Glyceraldehyde 3-phosphate dehydrogenase	Gapdh	XM_001473623	Forward: AAG AAG GTG GTG AAG CAG GCA TC Reverse: CGA AGG TGG AAG AGT GGG AGT TG
Sodium/*myo*-inositol transporter 1	Slc5a3	NM_017391	Forward: CTG TGG TGC TGT GGG ATG ATG Reverse: CCT GCT GGG TCT GAA CTT TGC
Sodium/*myo*-inositol transporter 2	Slc5a11	NM_146198	Forward: CAA GGT GGT GAG GGC TAT CC Reverse: CTA TGA CAG GTT CCG CTT TGC

### Substrate selectivity analysis

Human epithelial kidney (HEK293; ATCC, Cat #: CRL-1573) cells were cultured in DMEM medium containing 10% fetal bovine serum (FBS) and 1% penicillin/streptomycin. Cells were plated onto 24-well plates and grown to 90% confluency. Once 90% confluent, cells were washed in PBS (pH 7.0), then incubated in PBS containing: *scyllo*-inositol-(2-^3^H), *myo*-inositol-(2-^3^H) or L-fucose-(5,6-^3^H) (3 µCi/mL, 100 µM with 0.1% w/v BSA), with or without a substrate competitor. The substrate competitors (Figure 2) consisted of either inositol stereoisomers (*myo*-, *scyllo*-, *epi*-, *allo*-, *muco*-, *neo*-, L-*chiro*-, or D-*chiro*-inositol), inositol derivatives (viburnitol, D-ononitol, sequoyitol, L-quebinchitol or D-pinitol) or structurally similar sugar substrates (D-glucose, L-glucose, D-galactose, D-mannose, D-fucose, L-fucose, L-rhamnose, D-ribose, L-ribose or D-fructose). One well was dedicated to testing each competitor and triplicate plates were run for each experiment. Following incubation (3 h, 37°C), cells were washed twice with PBS containing 1 mM cold *myo*-inositol, to stop transporter activity, following which the cells were dissolved using 2% SDS and the radioactivity measured using a scintillation counter. Competitors were tested either at a fixed concentration (10 mM) or in a dose range (10–400 mM). Novel *scyllo*-inositol derivatives were used to test *myo*-inositol-(2-^3^H) transport requirements at a fixed concentration of 2 mM.

### Statistical analysis

Statistical analysis was conducted using the Statistical Analysis System (SAS) and the Graphpad Prism programs. Groups were compared using a one-way ANOVA. If a significant F score was observed (p<0.05), a Bonferroni post hoc test was used to compare groups with the statistical significance, p<0.05.

## Supporting Information

Figure S1 **A comparison of D- and L-** ***chiro*** **-inositol inhibition of myo-inositol transport in HEK293 cells.** Based on reduction in myo and scyllo-inositol transport by, D-*chiro*-inositol it is recognized by SMIT1/2, while L-*chiro*-inositol is not. This finding was examined more closely in HEK293 cells, by comparing the transport of *myo*-inositol-(2-^3^H) in the presence of increasing concentrations of D-*chiro*-inositol (**A**), to transport observed in the presence of increasing concentrations of L-*chiro*-inositol (**B**). As expected *myo*-inositol-(2-^3^H) transport was inhibited by D-*chiro*-inositol in a concentration dependent manner. In contrast, L-*chiro*-inositol did not inhibit *myo*-inositol-(2-^3^H) transport, except at the highest concentration, 160 mM. (n = 3 wells per variable).
(TIF)

Figure S2 **L-fucose-(5,6-^3^H) transport.** L-fucose was found to be a competitive inhibitor of *myo*- and *scyllo*-inositol-(2-^3^H) transport through SMIT1/2. This finding was further examined by: (**A**) examining *myo*-inositol-(2-^3^H) transport in the presence of increasing concentrations of L-fucose. A concentration-dependent reduction of *myo*-inositol-(2-^3^H) transport was observed. (**B**) An examination of L-fucose-(5,6-^3^H) transport in these cells, in the presence or absence of potential competitive substrates. Only background radioactivity was observed, with not active transport. (**C**) L-fucose-(5,6-^3^H) transport was examined in the presence or absence of D-fucose and increasing concentrations of cold L-fucose and again only background radioactivity was observed. (n = 3 wells per variable).
(TIF)
