# Supplementary figures and images for: Sodium/myo-Inositol Transporters: Substrate Transport Requirements and Regional Brain Expression in the TgCRND8 Mouse Model of Amyloid Pathology
Source: PLoS One. 2011 Aug 26;6(8):e24032. doi: 10.1371/journal.pone.0024032 (PMC3162603; doi:10.1371/journal.pone.0024032)

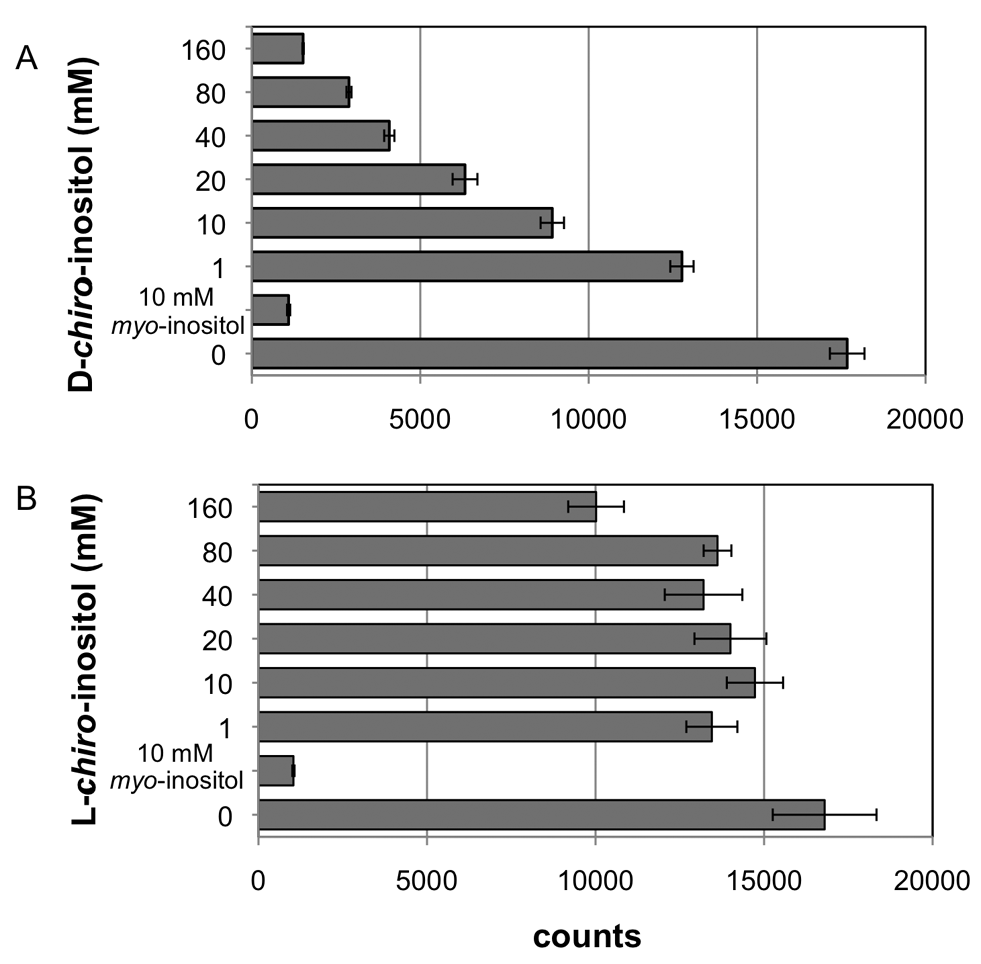

Supplement: Figure S1 — A comparison of D- and L- chiro -inositol inhibition of myo-inositol transport in HEK293 cells. Based on reduction in myo and scyllo-inositol transport by, D-chiro-inositol it is recognized by SMIT1/2, while L-chiro-inositol is not. This finding was examined more closely in HEK293 cells, by comparing the transport of myo-inositol-(2-3H) in the presence of increasing concentrations of D-chiro-inositol (A), to transport observed in the presence of increasing concentrations of L-chiro-inositol (B). As expected myo-inositol-(2-3H) transport was inhibited by D-chiro-inositol in a concentration dependent manner. In contrast, L-chiro-inositol did not inhibit myo-inositol-(2-3H) transport, except at the highest concentration, 160 mM. (n = 3 wells per variable). (TIF) [file pone.0024032.s001.tif]

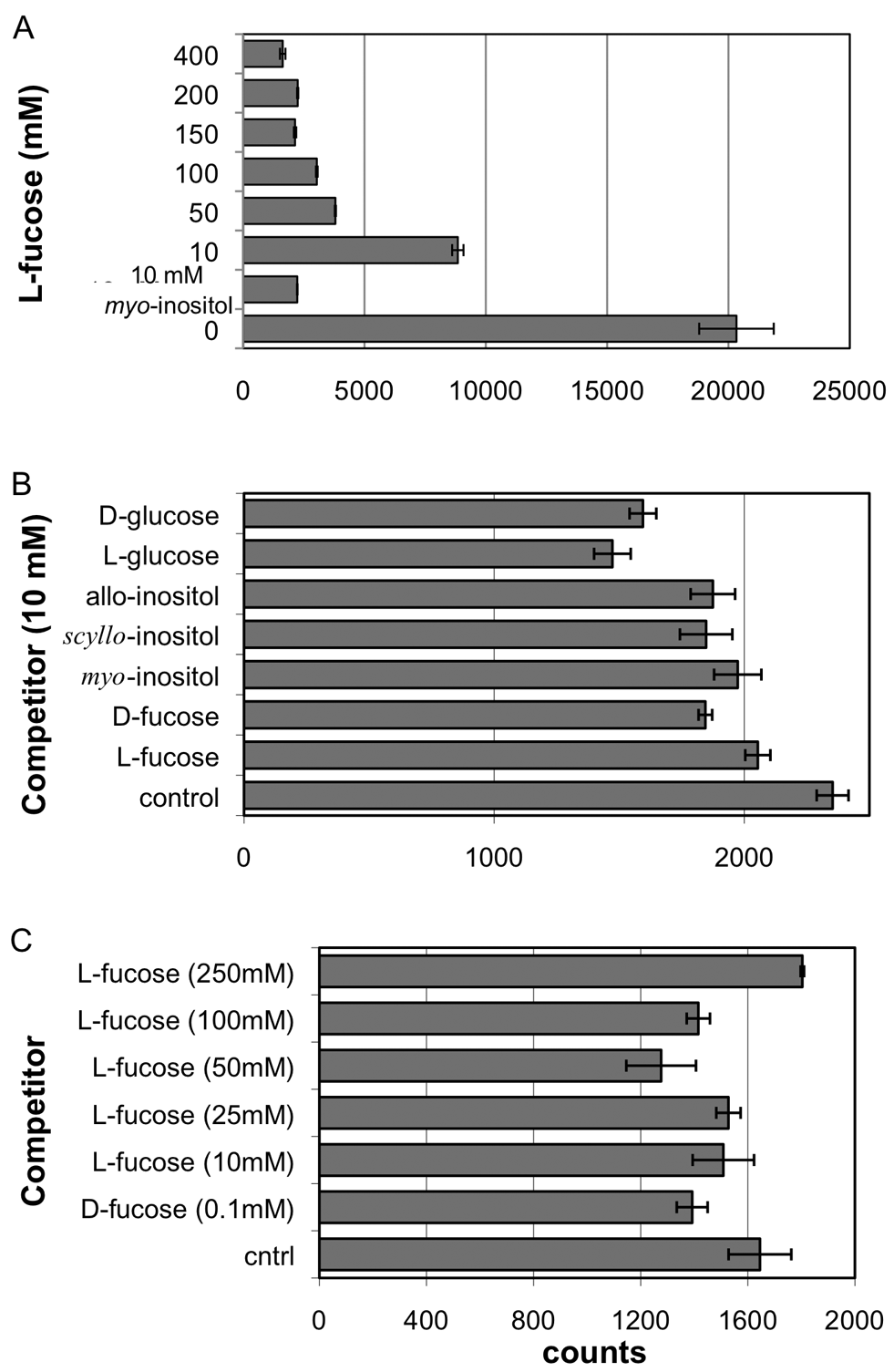

Supplement: Figure S2 — L-fucose-(5,6-3H) transport. L-fucose was found to be a competitive inhibitor of myo- and scyllo-inositol-(2-3H) transport through SMIT1/2. This finding was further examined by: (A) examining myo-inositol-(2-3H) transport in the presence of increasing concentrations of L-fucose. A concentration-dependent reduction of myo-inositol-(2-3H) transport was observed. (B) An examination of L-fucose-(5,6-3H) transport in these cells, in the presence or absence of potential competitive substrates. Only background radioactivity was observed, with not active transport. (C) L-fucose-(5,6-3H) transport was examined in the presence or absence of D-fucose and increasing concentrations of cold L-fucose and again only background radioactivity was observed. (n = 3 wells per variable). (TIF) [file pone.0024032.s002.tif]
